# Reply to ‘Evidence that neutrophils do not promote *Echis carinatus* venom-induced tissue destruction’

**DOI:** 10.1038/s41467-018-04507-y

**Published:** 2018-06-13

**Authors:** Kempaiah Kemparaju, Kesturu S. Girish, Gajanan D. Katkar

**Affiliations:** 1 Department of Studies in Biochemistry, University of Mysore, Manasagangothri, Mysuru, 570006 India; 2 Department of Studies and Research in Biochemistry, Tumkur University, Tumakuru, 572103 India; 3 Present Address: Institute of Biomedical Sciences, Academia Sinica, Taipei, 115 Taiwan

Prof. Reber and his team for providing further insight into *Echis carinatus* venom-induced tissue necrosis in their correspondence^1^ regarding our *Nature Communications* article^2^. We are glad that our major findings such as reduction in *E. carinatus* venom-induced tail injury after DNase I treatment, increased the mortality of mice when co-injected with *E. carinatus* venom and DNase I, function of venom DNase in toxicity, and the application of the newly developed mouse tail model to study sustained tissue necrosis have been ratified. These effects are due to the accumulation of extracellular DNA and its clearance by the DNase I treatment at the venom injected site. However, using a variety of neutropenic mouse models, the authors claim that neutrophils or neutrophils extracellular traps (NETs) do not contribute to *E. carinatus sochureki*, *E. carinatus multisquamatus,* or *E. carinatus pyramidum* venom-induced tissue necrosis, but the extracellular traps (ETs) derived from resident and other necrotic cells do contribute. Unlike our published article, they do not test *E. carinatus carinatus* (Indian saw-scaled viper) venom.

We believe that the differences in results are due to dose and species-dependent variation of the venoms tested. Snake venoms are highly complex mixtures, predominantly of enzymatic and non-enzymatic protein and peptide toxins that vary in lethal potency and pharmacological properties. Variability has been detected at various levels including inter-genus, inter-species, inter-subspecies, and within species and sub-species due to geographical or seasonal distribution, age, and diet^3–6^. Venom variability is thereby an intense area of research with serious implications for successful application of anti-venom therapy. In a previous study, we showed substantial differences in hemorrhage-inducing activity between *E. carinatus carinatus* and *E. carinatus sochureki*^7^, providing direct evidence of venom variability between *E. carinatus* sub-species. Furthermore, we believe it is probable that *E. carinatus sochureki*, *E. carinatus multisquamatus*, and *E. carinatus pyramidum* venoms used by the authors may vary among themselves.

We are glad that the authors acknowledge venom variability and that *E. carinatus carinatus* venom and the venoms that they have studied are different, and that this distinction might be responsible for the differences between the studies. In our study, both in vitro and in vivo data (assays of several markers of NETosis) demonstrate NETosis^2^. In contrast, the authors defend their finding using neutropenic mouse models injecting with a high dose of venom, but without defining the molecular mechanisms.

We believe that aside from venom variability, the differences between our studies may also result from a varied dose of venom injected. The authors have injected 3 mg venom/kg body weight in 25 µl, as against 1 mg venom/kg body weight in 50 µl injected in our study. Pertaining to the dose injected, although it appears that there exists a systemic difference of 1: 3 dose between the studies, actually, it is the difference between 1 mg in 50 μl vs. 3 mg in 25 μl at the injection site (mouse tail). Thus, it clearly suggests that there is a 1:6-fold increase in venom concentration at the injection site and that this might non-specifically lyse the resident and the other cells, including the subcellular membranes.

Regarding the criticism of our use of cyclophosphamide to achieve neutropenia, we agree that cyclophosphamide is a pleiotropic drug and affects various blood cells, including lymphocytes, monocytes, basophils, and eosinophils, as well as hematopoiesis itself. Although several studies have used cyclophosphamide to induce neutropenia^8, 9^, we resorted to the use of cyclophosphamide owing to a lack of access to other models of neutropenia.

## References

[1] Stackowicz, J. et al. Evidence that neutrophils do not promote *Echis carinatus*venom-induced tissue destruction. *Nat. Commun*. 10.1038/s41467-018-04688-6 (2018). 10.1038/s41467-018-04688-6 PMC5998045 29899337

[2] Katkar, G. D. et al. NETosis and lack of DNase activity are key factors in *Echis carinatus* venom-induced tissue destruction. *Nat. Commun*. **7**, 11361 (2016). 10.1038/ncomms11361 PMC4838891 27093631

[3] Barlow A, Pook CE, Harrison RA, Wüster W (2009). Coevolution of diet and prey-specific venom activity supports the role of selection in snake venom evolution. Proc. Biol. Sci..

[4] Shashidharamurthy R, Jagadeesha DK, Girish KS, Kemparaju K (2002). Variations in biochemical and pharmacological properties of Indian cobra (Naja naja naja) venom due to geographical distribution. Mol. Cell. Biochem..

[5] Chippaux JP, Williams V, White J (1991). Snake venom variability: methods of study, results and interpretations. Toxicon.

[6] Casewell, N. R., Harrison, R. A., Wuster, W. & Wagstaff, S. C. Comparative venom gland transcriptome surveys of the saw-scaled vipers (Viperidae: Echis) reveal substantial intra-family gene diversity and novel venom transcripts. *BMC Genomics* **10**, 564 (2009). 10.1186/1471-2164-10-564 PMC2790475 19948012

[7] Sunitha K (2011). Neutralization of haemorrhagic activity of viper venoms by 1-(3-dimethylaminopropyl)-1-(4-fluorophenyl)-3-oxo-1,3-dihydroisobenzofuran-5-carbonitrile. Basic Clin. Pharmacol. Toxicol..

[8] Zuluaga AF (2006). Neutropenia induced in outbred mice by a simplified low-dose cyclophosphamide regimen: characterization and applicability to diverse experimental models of infectious diseases. BMC Infect. Dis..

[9] Dale DC, Alling DW, Wolff SM (1972). Cyclic hematopoiesis: the mechanism of cyclic neutropenia in grey collie dogs. J. Clin. Invest..

